# Investigating capillary electrophoresis‐mass spectrometry for the analysis of common post‐translational modifications

**DOI:** 10.1002/elps.201700437

**Published:** 2018-03-08

**Authors:** Klaus Faserl, Bettina Sarg, Peter Gruber, Herbert H. Lindner

**Affiliations:** ^1^ Division of Clinical Biochemistry Biocenter Innsbruck Medical University Innsbruck Tirol Austria; ^2^ Division of Medical Biochemistry Biocenter Innsbruck Medical University Innsbruck Tirol Austria

**Keywords:** CE‐MS, Neutral coating, Phosphorylation, Post‐translational modification

## Abstract

Capillary electrophoresis coupled to mass spectrometry is a very efficient analytical method for the analysis of post‐translational modifications because of its high separation efficiency and high detection sensitivity. Here we applied CE‐MS using three differently coated separation capillaries for in‐depth analysis of a set of 70 synthetic post‐translationally modified peptides (including phosphorylation, acetylation, methylation, and nitration). We evaluated the results in terms of peptide detection and separation characteristics and found that the use of a neutrally coated capillary resulted in highest overall signal intensity of singly modified peptides. In contrast, the use of a bare‐fused silica capillary was superior in the identification of multi‐phosphorylated peptides (12 out of 15 were identified). Fast separations of approximately 12 min could be achieved using a positively coated capillary, however, at the cost of separation efficiency. A comparison to nanoLC‐MS revealed that multi‐phosphorylated peptides interact with the RP material very poorly so that these peptides were either washed out or elute as very broad peaks from the nano column which results in a reduced peptide identification rate (7 out of 15). Moreover, the methods applied were found to be very well suited for the analysis of the acetylated, nitrated and methylated peptides. All 36 synthetic peptides, which exhibit one of those modifications, could be identified regardless of the method applied. As a final step in this study and as a proof of principle, the phosphoproteome enriched from PC‐12 pheochromocytoma cells was analyzed by CE‐MS resulting in 5686 identified and 4088 quantified phosphopeptides. We compared the characterized analytes to those identified by a nanoLC‐MS proteomics study and found that less than one third of the phosphopeptides were identical, which demonstrates the benefit by combining different approaches quite impressively.

**Abbreviations**
BGE: Background electrolyte
CE‐MS: Capillary electrophoresis ‐ mass spectrometry
RP‐HPLC: Reversed phase ‐ high performance liquid chromatography

## 1 Introduction

Mass spectrometry is the method of choice for the analysis of post‐translational modifications (PTM's) on proteins. An enzymatic cleavage of proteins into peptides followed by either LC‐MS or CE‐MS analysis is preferably performed to identify those peptides that carry modifications. To get the tremendous complexity of biological samples under control and to increase signal intensity of modified peptides an enrichment step is frequently implemented into the proteomics workflow. This can either be done by decreasing the complexity of the sample and analyzing a single or a set of proteins with particular attention, or by selectively enriching peptides with a specific modification, e. g., phosphopeptides.

Despite latest instrument improvements, PTM analysis is still challenging. The problems are complex and can occur throughout the entire proteomics workflow, e. g. for the majority of modifications a specific enrichment method is not available; the enrichment method is often not fully specific; modified peptides are frequently present in lowest quantities, and co‐eluting substances provoke ion suppression effects that prevent peptide detection.

In this context, analyte separation by capillary electrophoresis prior to MS analysis can offer substantial benefits. Since the reason for the introduction of many post‐translational modifications is the alteration of proteinstructure and a change in their biological activity, the modification is often accompanied by a change in the charge state of the modified amino acid residue 1, 2. For example, phosphorylation introduces a group with an acidic pKa thus having a great impact on the p*I* of a given peptide. Similarly, modifications such as acetylation, which cause the amino acid residue to change from a basic residue to a neutral residue, will also have great impact. Effectively, a larger pKa alteration results in a larger change in peptide p*I* and therefore a larger shift of the electrophoretic mobility of the peptide in CE. These changes in peptide p*I* were recently utilized to analyze asparagine and glutamine deamidation and isomerization and arginine citrullination of myelin basic proteins (MBP´s) in mice of different ages 3. Due to significant differences in the pKa values of aspartate and isoaspartate and of glutamate and isoglutamate, respectively, these isomerized forms could be assigned based on a shift in migration time. In a similar manner peptide and protein deamidation has been investigated in the course of peptide drug analysis 4, the analysis of a protein standard mixture 5, and in the course of monoclonal antibody studies 6, 7, 8. Even site‐specific effects can influence the overall charge of peptides thus causing a CE separation of phosphopeptides positional isomers 5.

A mayor advantage of capillary electrophoresis is the ability to work at very low flow rates. It has been shown by direct infusion experiments of angiotensins, that a flow rate reduction yields higher detection sensitivity in MS 9. This was also found to be true for the analysis of phosphopeptides, whose detection sensitivity was strongly enhanced at flow rates below 30 nL/min 10. A further reduction down to 10 nL/min, can be feasible by combining a sheathless interfaces with a neutrally coated capillary 11. This setup was successfully utilized e. g. in the course of proteomics studies, for phosphopeptide and for glycopeptide analysis 10, 11, 12, 13.

The aim of this study was to investigate the migration behavior of peptides modified with common PTM´s and to evaluate different coatings for their analysis. Three capillaries with different inner surfaces, a neutrally coated, a positively coated (modifier used was polyethyleneimine), and a bare‐fused silica capillary were used. We investigated a set of 70 synthetic posttranslationally modified peptides that allowed us to analyze a variety of modifications, with a special focus on phosphorylation but also acetylation, methylation, and nitration. For peptide ionization and detection we used the sheathless CE‐MS interface distributed by Sciex and a Thermo LTQ Orbitrap XL mass spectrometer. Furthermore, as a proof of principle, the phosphoproteome of PC‐12 pheochromocytoma cells was quantified by CE‐MS. To expand the phosphoproteome coverage and to benchmark the CE‐MS workflow, we analyzed the same sample using a complementary approach utilizing nanoLC‐MS. Characteristic features of the two approaches and the number of identified and quantified phosphopeptides were compared.

## 2 Materials and methods

### 2.1 Materials

Synthetic posttranslationally modified peptides were kindly provided by Alexander R. Ivanov. A similar mixture of these synthetic modified peptides was used during the ABRF‐sPRG Study (Association of Biomolecular Resource Facilities ‐ Proteomic Standards Research Group) in 2011–2012. A total of 70 synthetic peptides were analyzed representing a variety of modifications, including acetylation, methylation, nitration, and phosphorylation.

### 2.2 Capillary electrophoresis: mass spectrometry (CE‐MS)

For peptide separation and ionization a PA 800 plus capillary electrophoresis system (Sciex, Brea, CA) was coupled via an ESI module to a Thermo LTQ Orbitrap XL mass spectrometer (Bremen, Germany). The capillary electrophoresis system was equipped with a fused‐silica capillary with a porous tip acting as nanospray emitter (total length: 100 cm, i.d.: 30 μm, o.d.: 150 μm). Three types of separation capillaries were used: (i) a bare fused‐silica capillary, (ii) a neutral capillary (the coating comprises two layers, a hydrophobic coating and a hydrophilic polyacrylamide coating; manufactured by Sciex), and (iii) a capillary with a positively charged surface (modifier used was polyethyleneimine (PEI)). Prior to each analysis the system was rinsed with background electrolyte for 3 min at 50 psi. For separations using the bare and neutrally coated fused‐silica capillaries 10% (v/v) acetic acid was used as BGE and +30 kV as separation voltage. For separations using the neutral capillary a simultaneous pressure of 1 psi was applied at the capillary inlet. The positively coated capillary was operated using 0.1% (v/v) formic acid as BGE and −25 kV as separation voltage.

### 2.3 Mass spectrometry data acquisition

The LTQ Orbitrap XL mass spectrometer was operating in positive ion mode applying a data dependent automatic switch between survey scan and MS/MS acquisition. Survey full scan MS spectra were acquired in the Orbitrap with a resolution of R = 30000 at an AGC target of 1e6 and 50 ms maximum ionization time. To generate MS/MS spectra the three highest precursors were selected for collision‐induced dissociation (CID) in the linear trap applying normalized collision energy of 35.0. Fragments were scanned in the linear trap in cntroide mode at an AGC target of 2e5. Dynamic exclusion settings were as follows: repeat count was 2; repeat duration was 3 s; exclusion duration was 30 s; unassigned and singly charged peptides were excluded from CID fragmentation. A neutral loss of 49, 32.66 or 24.5 detected in one of the three most intense ions in MS² was decisive for a MS³ which was performed to reaffirm the peptide sequence.

### 2.4 Data analysis and protein identification

Proteome Discoverer version 1.4 (ThermoScientific) with search engine Sequest was used for data analysis. MS/MS spectra were searched against a FASTA database including target proteins and typical contaminant proteins (2.001 entries) using the following settings: Enzyme for protein cleavage was trypsin; two missed cleavages were allowed. Variable modifications were phosphorylation of Ser, Thr, and Tyr, nitrosylation of Tyr, acetylation of Lys, mono‐ and di‐methylation of Lys and Arg, tri‐methylation of Lys, oxidation of Met, and deamidation of Asn. Precursor mass tolerance was set to 10 ppm. The fragment mass tolerance was 0.5 Da. False discovery rate (FDR) for proteins and peptides was set to 1%.

### 2.5 Quantitatice phosphoproteomics using CE‐MS

Materials and methods for the Quantitatice Phosphoproteomics analyses of SILAC labeled PC‐12 pheochromocytoma cells can be found in the Supporting Information.

## 3 Results and discussion

### 3.1 Phosphopeptides in capillary electrophoresis

To analyze migration behavior of posttranslational modified peptides in detail, we investigated a synthetic peptide mixture containing a total of 70 differentially modified peptides. Amongst them were: 14 mono‐phosphorylated and 15 multi‐phosphorylated peptides (5 di‐, 6 tri‐, and 4 tetra‐phosphorylated peptides), 26 methylated (10 mono, 11 di and 5 tri‐methylated peptides), 5 acetylated, and 5 nitrated peptides. Due to the importance of protein phosphorylation in cell signaling, e. g. to activate or inhibit enzyme activity, we were mainly interested in the analysis of phosphorylated peptides present in this sample. Protein phosphorylation introduces a hydrophilic group and, most importantly for separations performed by capillary electrophoresis, the introduced phosphate group possesses a negative charge, which reduces the overall net charge of the peptide causing a decrease in its electrophoretic mobility under the pH conditions chosen (pH 2.3 or pH 2.7). Therefore, phosphopeptides migrate significantly slower than their unmodified counterparts resulting in a full separation by CE. In addition to that, the low‐ or ultra‐low flow conditions typical for CE separations significantly reduce ion suppression effects permitting a highly sensitive identification of phosphopeptidides.

**Figure 1:**
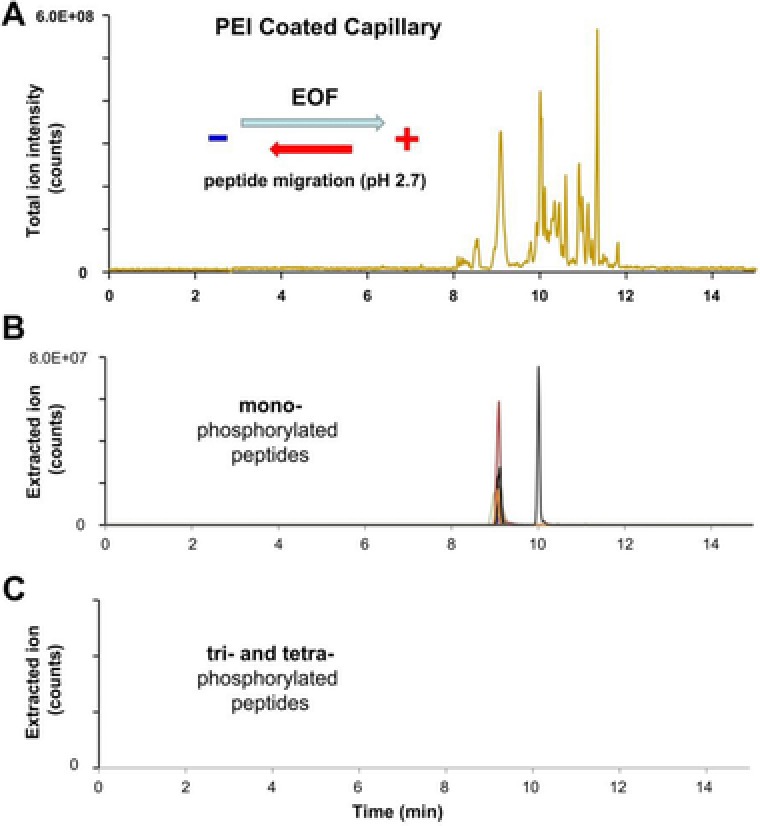
CE‐MS separation of post‐translational modified synthetic peptides using a PEI coated capillary. (A) Total ion current electropherogram and extracted ion electropherograms of (B) mono‐phosphorylated and (C) tri‐ and tetra‐phosphorylated peptides. CE conditions: Separation capillary length: 100 cm with porous tip, i.d.: 30 μm, o.d.: 150 μm; BGE: 0.1% (v/v) formic acid; separation voltage:  −25 kV.

In the course of this study, we investigated the migration behavior of the modified peptides using three different types ofcapillaries, (i) a positively charged, (ii) a bare fused, and (iii) a neutrally coated capillary . The first capillary we used was treated with polyethyleneimine (PEI), a modifier that causes the inner capillary surface originally negatively charged to be positively charged. For CE separations, we applied a voltage of ‐25 kV (reversed mode) and used 0.1% formic acid (pH 2.7) as BGE. These separation conditions result in a very high electroosmotic flow (> 100 nL/min; towards the anode) that carries the peptides to the inlet of the MS instrument, whereas the positively charged peptides migrate towards the cathode, thus to the opposite direction. This setup works perfectly as long as the magnitude of the EOF is higher than the electrophoretic mobility of the analytes, which is true for almost all peptides. As depicted in the total ion current electropherogram in Fig. 1A, fast separations of approximately 12 min can be achieved using PEI coated capillaries. The time frame for peptide detection was roughly 4 min, starting at minute 8. Due to the reduced electrophoretic mobility of the phosphorylated peptides, they enter the mass analyzer first, just before the unmodified analytes. Database search was able to identify 12 out of 29 phosphopeptides, with 11 mono‐ and a single di‐phosphorylated one. As can be seen in Fig. 1B, the mono‐phosphorylated peptides co‐migrate at minute 9. The difference in their mobility is insufficient for their separation within this fast analysis. Sole exception is THILLFLPKpSVSDYEGK, since this phosphopeptide comprises one additional basic amino acid that increases the peptide´s electrophoretic mobility significantly. Therefore, this peptide was detected later, at minute 10. Interestingly, no tri‐ or tetra‐phosphorylated peptide could be detected, neither by database search nor by manual extraction of ions (Fig. 1C).

We first assumed ion suppression effects causing their absence since these exceptionally slow migrating peptides may not leave the sample zone. However, ionic analytes typically causing ion suppression effects should have left the sample zone by the time it reaches the mass spectrometer. Since the ionization efficiency of multiply phosphorylated peptides strongly depends on the flow rates at which ionization occurs 9, a more likely explanation for their absence is that the efficiency to ionize such species is too low under the separation conditions used.

**Figure 2:**
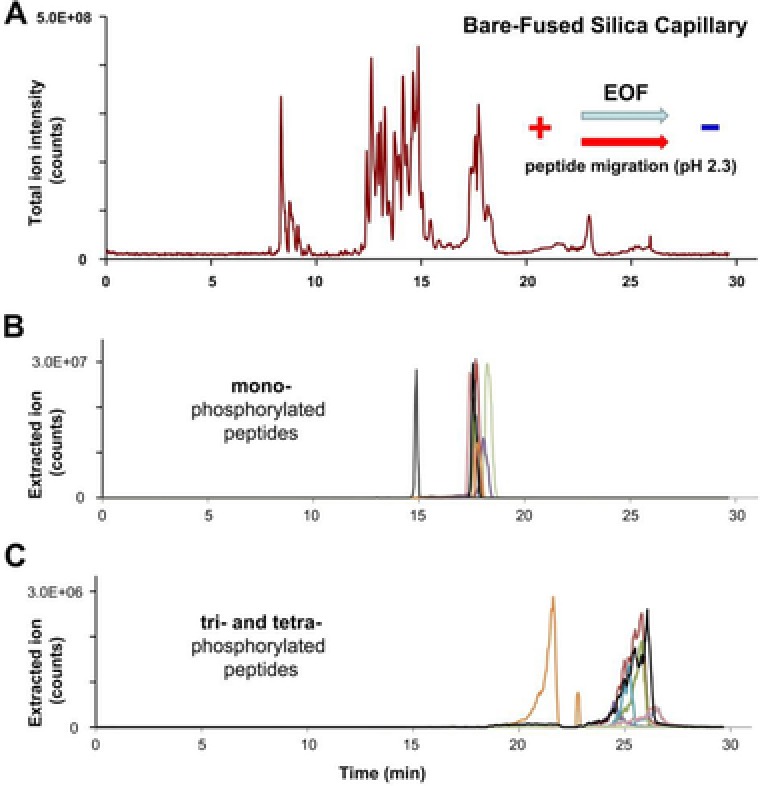
CE‐MS separation of post‐translational modified synthetic peptides using a bare‐fused silica capillary. (A) Total ion current electropherogram and extracted ion electropherograms of (B) mono‐phosphorylated and (C) tri‐ and tetra‐phosphorylated peptides. CE conditions: Separation capillary length: 100 cm with porous tip, i.d.: 30 μm, o.d.: 150 μm; BGE: 10% (v/v) acetic acid; separation voltage: +30 kV.

Despite the fact that the PEI coating permits very fast analyses, the narrow separation window of PEI coated capillaries can be considered the downside of this setup, especially for the analysis of more complex mixtures. To overcome this disadvantage, a bare‐fused silica capillary and 10% (v/v) acetic acid (pH 2.3) was used as BGE for further separation studies. The lower pH of this BGE minimizes both, possible interactions of peptides with the capillary surface and the magnitude of the EOF, which is pH dependent and rises rapidly above pH = 4 14. When using a bare‐fused silica capillary the capillary surface is negatively charged and the resulting EOF directed to the cathode. Therefore, the instrument has to be used in “normal mode” and the migration order of the peptides is opposite to that obtained with a PEI coated capillary (Fig. 2A). Twelve mono‐phosphorylated peptides were detected between minutes 17 and 18.5 (the peptide THILLFLPKpSVSDYEGK was detected at minute 15), approximately 2 min after the non‐phosphorylated peptides (Fig. 2B). In stark contrast to the previous result, database search identified 4 out of 5 di‐phosphorylated peptides, all 6 tri‐phosphorylated peptides, and 2 out of 4 tetra‐phosphorylated peptides. Interestingly, many tri‐ and tetra‐phosphorylated peptides were detected behind the injection plug after minute 23 (Fig. 2C), which means that these analytes must possess a negative net charge. When calculating the isoelectric point of the phosphopeptides the impact of a phosphate group becomes apparent: peptide TITLEVEPSDTIENVK modified with two phosphate groups exhibits a p*I* of 2.86 compared to a p*I* of 2.09 when modified with four phosphate groups. Due to the 10% (v/v) acetic acid (pH 2.3) used as BGE the di‐phosphorylated peptide has still a positive net charge and migrates toward the cathode, whereas the tetra‐phosphorylated analyte is negatively charged and migrates into the opposite direction toward the anode. The overall separation window could be increased from 4 min using the PEI coated capillary to approximately 15 min using the BFS capillary. The rather concentrated samples analyzed in the course of this study resulted in a deterioration of phosphopeptide peak shapes which was especially true for multiply‐phosphorylated peptides shown in Fig. 2C. In a further experiment we diluted the sample and injected 6 fmol of sample resulting in improved peak shapes; however at the cost of identification rate (data not shown).

**Figure 3:**
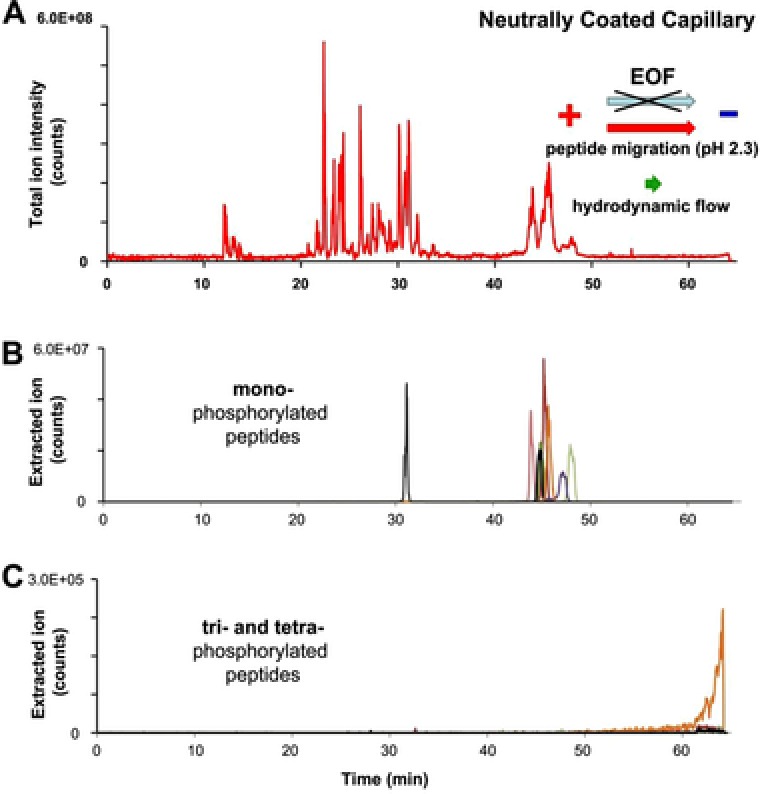
CE‐MS separation of post‐translational modified synthetic peptides using a neutrally coated capillary. (A) Total ion current electropherogram and extracted ion electropherograms of (B) mono‐phosphorylated and (C) tri‐ and tetra‐phosphorylated peptides. CE conditions: Separation capillary length: 100 cm with porous tip, i.d.: 30 μm, o.d.: 150 μm; BGE: 10% (v/v) acetic acid; separation voltage: +30 kV with a pressure of 1 psi applied at the capillary inlet.

To complete this study, we investigated the use of a neutrally coated capillary as a significant increase in phosphopeptides signal intensity was found when working at very low flow rates of about 10 nL/min 10. A neutrally coated capillary, as its name suggests, exhibits an inner capillary surface with a net charge of almost zero and completely suppresses EOF. To obtain a stable ESI spray using the sheathless interface, a flow of liquid must be generated in the separation capillary by applying a pressure of 1 psi at the capillary inlet. Due to the low flow rate generated (5–10 nL/min), peptide migration is primarily determined by the electrophoretic mobility of the peptide itself, thus enabling highest separation selectivity. As can be seen in the total ion current electropherogram (Fig. 3A), the separation window could be enlarged to more than 40 min with simultaneous increase of separation selectivity. The mono‐phosphorylated peptides migrate in the range between minute 44 and 49. These peptides could be separated significantly better compared to previous results and showed higher overall signal intensity, which is in accordance to previously published data. 9, 10. As a result, database search yielded 12 mono‐, 4 di‐, and 1 tri‐phosphorylated peptide. Due to the low flow rate and the fact that multi‐phosphorylated peptides migrate very slowly or even towards the capillary inlet, peptides with three or four phosphate groups do not reach the MS instrument, and thus cannot be analyzed under these separation conditions. The raise in intensities shown in Fig. 3C at the end of the separation indicates the migration of a single tri‐ phosphorylated peptide. To push other multiply phosphorylated peptides toward MS instrument a higher flow rate in the capillary would be needed throughout the entire separation, which is possible when applying a higher pressure at the capillary inlet, however, with the downside of losing separation selectivity and most probably signal intensity. When applying a pressure towards the end of the CE‐run no additional peptides could be detected.

### 3.2 Phosphopeptides in high‐performance liquid chromatography

**Figure 4:**
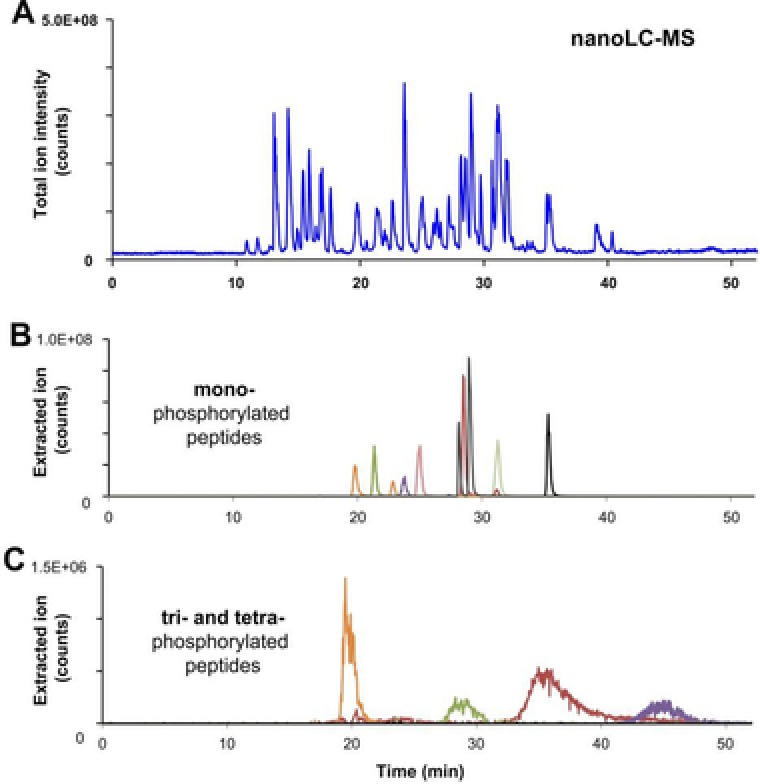
nanoLC‐MS separation of post‐translational modified synthetic peptides using a neutrally coated capillary. (A) Total ion current chromatogram and extracted ion chromatograms of (B) mono‐phosphorylated and (C) tri‐ and tetra‐phosphorylated peptides. LC‐MS was performed using a homemade fritless column; packed 10 cm with 3 μm reverse‐phase C18 (Reprosil). The gradient (solvent A: 0.1% formic acid; solvent B: 0.1% formic acid in 85% acetonitrile) started at 4%B and was increased linearly to 50% during 50 min and to 100% during 5 min. A flowrate of 250 nL/min was applied.

To compare the outcome with HPLC, a nanoLC‐MS analysis was performed using a homemade fritless column packed 10 cm with 3 μm reverse‐phase C18 resin. In Fig. 4A, the total ion current chromatogram of the synthetic peptide mixture is presented. As the primary advantage of LC over CE is the much higher mass loading capability, more than 4 times more sample was loaded; 130 fmol in LC compared to 30 fmol injected in CE.

**Figure 5:**
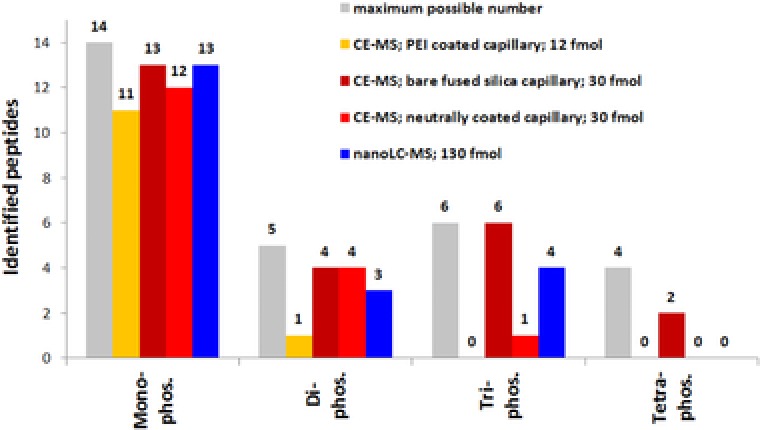
Summary of phosphopeptides identified during method evaluation. A total of 29 phosphopeptides present in the synthetic peptide mixture were analyzed by nanoLC‐MS and by CE‐MS using three differently coated separation capillaries (PEI coated, neutrally coated and bare‐fused silica capillary).

Database search of LC‐MS fragment spectra resulted in the identification of 13 mono‐, 3 di‐, and 4 tri‐phosphorylated peptides, which is less than obtained by CE‐MS using the bare‐fused silica capillary (Fig. 5). Both methods were able to identify the same number of 13 mono‐phosphorylated peptides whereas CE‐MS was superior in identifying multiply phosphorylated peptides as these tend do elute as very broad peaks in LC‐MS (Fig. 4C). Some multiply phosphorylated peptides could not be detected at all; probably this group of peptides poorly interacts with the RP material and, therefore, was washed out.

### 3.3 Acetylated, methylated, and nitrated peptides

**Figure 6:**
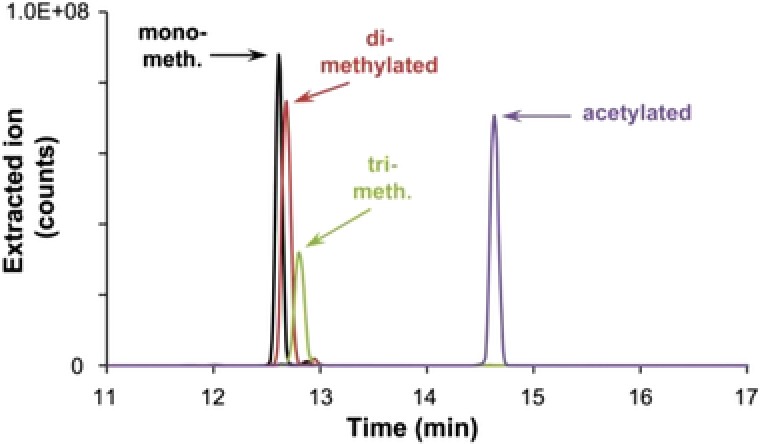
Extracted ion electropherograms of methylated and acetylated forms of peptide LKAEGSEIR analyzed by CE‐MS using a bare fused silica capillary. Ions were extracted from the total ion current electropherogram shown in Fig. 2. CE conditions as described in Fig. 2.

The migration behavior of the 5 acetylated, 26 methylated, and 5 nitrated peptides present in the synthetic peptide mixture was also investigated. Database search of the three different CE‐MS analysis revealed the identification of all 36 synthetic peptides in each experiment. In LC‐MS 35 modified peptides could be identified, interestingly a single acetylated peptide was not found since the corresponding ions were not present in the extracted ion chromatogram. As an example, the impact of different modifications on migration behavior is shown for peptide LKAEGSEIR (Fig. 6). Using a bare‐fused silica capillary the mono‐ and di‐methylated peptides differ in migration time by 4.5 s, whereas the di‐ and tri‐methylated forms differ by 7.9 s. The acetylated peptide enters the mass analyzer 1.84 min after the tri‐methylated form. Due to the almost identical masses of tri‐methylation (+42.047 Da) and acetylation (+42.010 Da), the pre‐separation of those forms is essential for unambiguous identification. This has previously been shown for the analysis of histones as this protein family exhibits multiple acetylation and methylation sites 15, 16, 17, 18, 19. No significant difference was found for the identification of nitrated peptides by LC‐ and CE‐MS; both methods are particularly well suited for their analysis. A summary of the identified peptides including migration time and peptide identification score can be found in the Supporting Information Table 1.

### 3.4 Quantitative phosphoproteomics using CE‐MS

In the course of a kinase activity study phosphopeptides enriched by IMAC from PC‐12 pheochromocytoma cells, a widely used model system for neuronal studies, were analyzed by CE‐MS and LC‐MS. The cells were grown under three different biological conditions and in presence of isotopically labeled and unlabeled lysine and arginine: Lys‐0 and Arg‐0; Lys‐4 and Arg‐6; or Lys‐8 and Arg‐10. To quantify changes in protein phosphorylation the two approaches that identified most phosphopeptides during method evaluation, CE‐MS using the bare‐fused silica capillary and nanoLC‐MS were applied.

**Figure 7:**
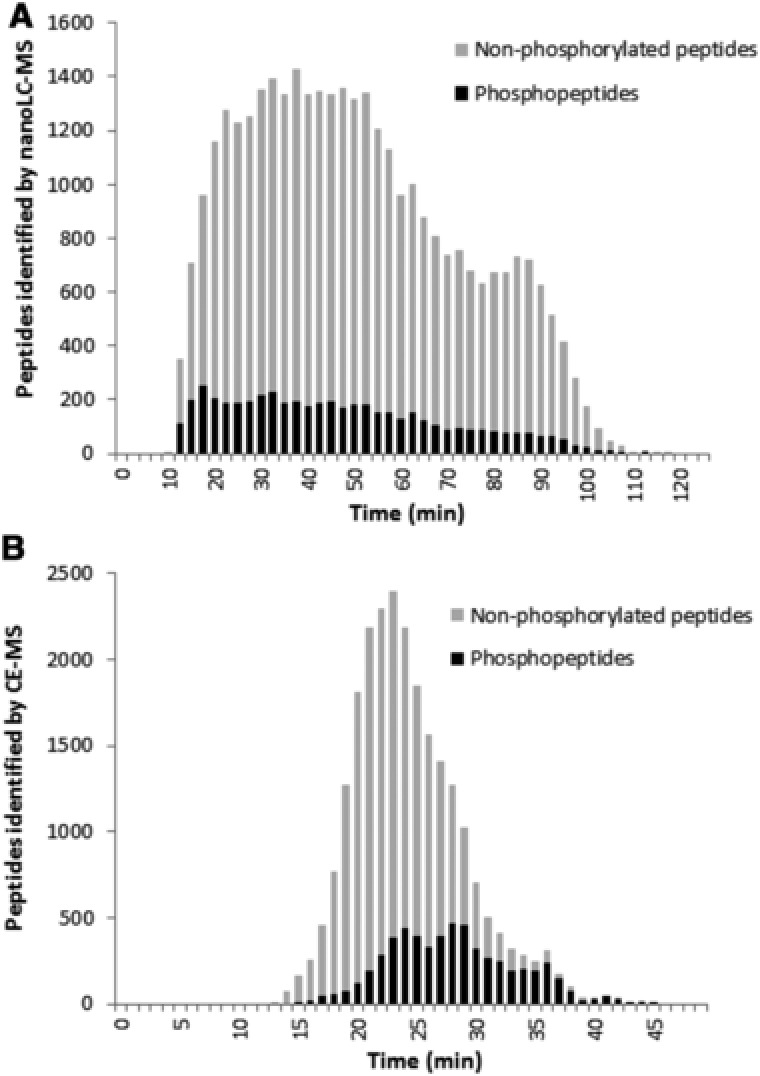
(A) Elution profile of 5006 phosphorylated and 29268 non‐phosphorylated peptides in 30 nanoLC‐MS analyses, shown as an overlay. (B) Migration profile of 5686 phosphorylated and 29268 non‐phosphorylated peptides identified in 103 CE‐MS analyses.

The first strategy for nanoLC comprised in‐solution protein digestion followed by phosphopeptide enrichment (IMAC) and a high pH reversed phase HPLC pre‐fractionation. This strategy is based on the two‐dimensional HPLC approach described by Richard D. Smith 20, 21. To compensate the lack in complementarity of the two dimensions and to reduce the overall analysis time we pooled an early, middle, and late eluting HPLC fraction previous to LC‐MS analysis. Due to the low scanning speed of the MS instrument used, a total of 30 nanoLC‐MS analysis were performed and yielded 5006 identified phosphopeptides. The total analysis time was 67 h; the data acquisition time of the mass spectrometer was 60 h. A set of 3840 phosphopeptides could be quantified spanning an intensity range of around 4.5 orders of magnitude (1.7·10^6^ to 7.4·10^9^ counts per peptide). As expected when using IMAC enrichment, a high number of 29 268 non‐phosphorylated peptides was present (Fig. 7A). Therefore we assume that coeluting non‐phosphorylated peptides still present after IMAC enrichment deteriorated phosphopeptide detection due to ion suppression effects. The intensities of the non‐phosphorylated peptides (2.1·10^6^ to 8.0·10^9^ counts per peptide) were in the same range as those for the phosphopeptides.

To expand the phosphoproteome coverage, we analyzed the same sample applying an alternative fractionation strategy, which was recently investigated in detail for its use in shotgun proteomics 11. The initial strategy comprised a reversed phase HPLC fractionation at acidic pH followed by ultralow flow CE‐MS analysis for high efficient peptide separation. We modified the original approach by introducing a phosphopeptide enrichment step before HPLC fractionation and by replacing the neutrally coated capillary with a bare fused silica capillary. Due to the low scanning speed of the MS analyzer 103 fractions were analyzed by CE‐MS. Total analysis time was 58.4 h; data acquisition time of the mass spectrometer was 51.5 h and therefore significantly shorter compared to the LC‐MS approach. However, the two approaches were not optimized to obtain the maximum number of peptides within the shortest possible time. Database search of acquired CE‐MS data resulted in a total of 5686 identified and 4088 quantified phosphopeptides. The number of non‐phosphorylated peptides was found to be 18 528. Compared to the LC‐MS approach, 37% less non‐phosphorylated peptides but 17.5% more quantified phosphopeptides were obtained.

Interestingly, despite the higher yield by CE‐MS, the overall phosphopeptides signal intensity was higher in nanoLC‐MS. The median signal intensity of phosphopeptides detected by CE‐MS was 2.03·10^7^ counts compared to 1.05·10^8^ counts in nanoLC‐MS. This means a roughly 5‐fold higher signal in nanoLC‐MS. An explanation for the yet higher number of phosphopeptides found in CE‐MS (despite the lower signal intensity) might be the separation of phosphopeptides from the large number of unmodified peptides. Due to the influence of the phosphate group on the electrophoretic mobility the majority of phosphopeptides migrate significantly slower than the unmodified peptides present in the same sample. This results in a lower complexity of the mass spectra during phosphopeptide detection and most likely in a decrease in ion‐suppression effects (Fig. 7B). Both effects increase the identification rate.

**Figure 8:**
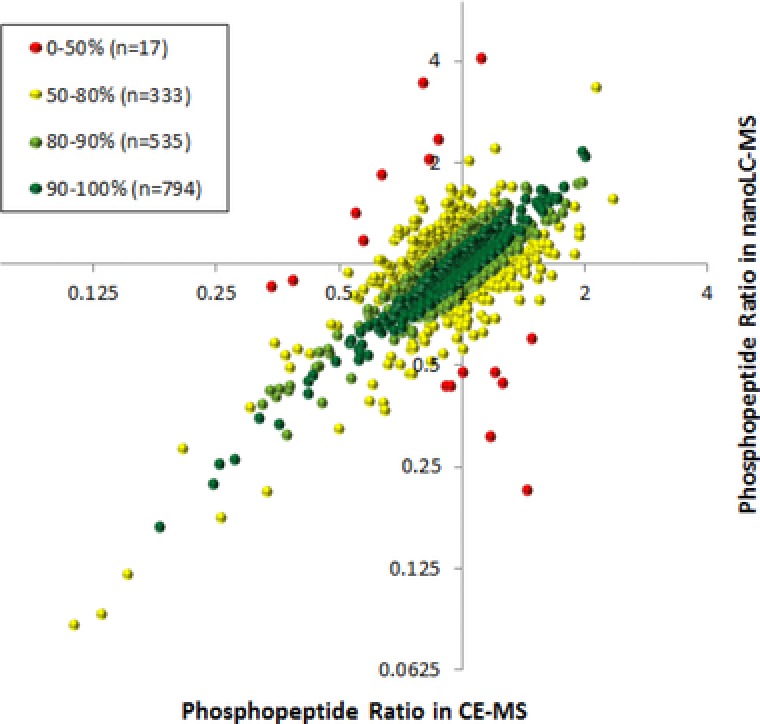
Reproducibility of phosphopeptide H/M ratios obtained by the two complementary approaches. Each dot represents a single phosphopeptide identified and quantified by both approaches. Illustrated dots were labeled according to the consistency of ratios obtained by the two complementary approaches given in the insert.

To evaluate the outcome of the two biological replicates with regard to phosphopeptide quantification, we compared the SILAC ratios obtained by both strategies. In the interest of simplification, only H/M ratios were used for data evaluation. The H/M ratios of all 1679 phosphopeptides quantified by both approaches were plotted against each other (Fig. 8). In addition, the cross ratios of individual protein H/M ratio pairs were calculated resulting in values ranging from 0 up to 1, corresponding to 0–100% consistency in quantification (shown as an insert in Fig. 8). The cross ratio calculation revealed high consistency between both approaches with 794 peptides agreeing in their H/M ratio better than 90%. An additional set of 535 peptides ranged between 80 and 90% and only 17 peptides were below 50%.

**Figure 9:**
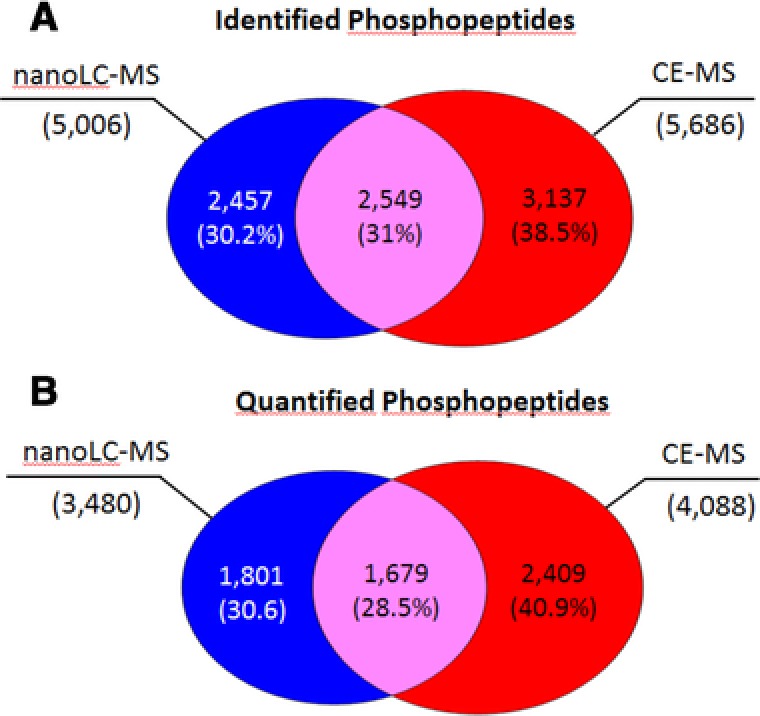
Venn diagram showing the overlap of (A) identified and (B) quantified phosphopeptides applying the nanoLC‐MS and CE‐MS approach. A total of 8143 phosphopeptides could be identified and thereof 5889 were quantified.

In previous studies 15, 22 we found that peptides of low molecular mass were preferentially identified by CE‐MS, which was subsequently confirmed by other groups. Furthermore, it has been shown that rather basic and hydrophilic peptides are also problematic to be analyzed by nanoLC‐MS 23, 24. In contrast, the higher sample amount that can be loaded onto the nanoLC system enables the detection of low abundance peptides by nanoLC‐MS only. With this in mind, we investigated the complementarity of the two approaches. The combined results revealed that the two approaches are able to identify 8143 phosphopeptides with only 2549 peptides overlapping. According to Fig. 9A, 2457 peptides were identified in the nanoLC‐MS and 3137 in the CE‐MS dataset only. The distribution of the quantified phosphopeptides follows closely the pattern of the identified ones with a total of 5889 quantified peptides and again a very low overlap of less than 30% (Fig. 9B). These data clearly show that more phosphorylated peptides could be characterized by the CE‐MS approach but more importantly, the two techniques complement each other in a perfect manner. The combination of both approaches increased phosphopeptide quantification by 69% demonstrating the advantage of combining both approaches for comprehensive phosphoproteome analysis.

## 4 Conclusion

In this study, we evaluated the suitability of CE‐MS for the analysis of post‐translationally modified peptides with special focus on the capillary modifier used. The results clearly demonstrate that CE‐MS is well suited for the analysis of modified peptides since more modified peptides were found by CE‐MS compared to nanoLC‐MS. But attention has to be paid on the capillary used for separation. For example the neutrally coated capillary resulted in highest overall signal intensity of singly modified peptides and provided highest separation selectivity. Whereas, a bare‐fused silica capillary was superior in identification of multiply phosphorylated peptides.

For phosphoproteome analysis of PC‐12 cells, the CE‐MS method was compared with nanoLC‐MS. Phosphopeptide quantification revealed very high consistency in peptide ratios between both approaches confirming their suitability for relative quantification using SILAC. Within roughly the same analysis time, the CE‐MS based approach was able to quantify more phosphopeptides compared to the LC‐MS approach. However, the combination of both approaches increased the outcome significantly demonstrating the advantage of combining both approaches for comprehensive phosphoproteome analysis.

*The authors have declared no conflict of interest*.

## Supporting information

Supporting information

Supporting information
