# Supplementary material for: Investigating capillary electrophoresis‐mass spectrometry for the analysis of common post‐translational modifications
Source: Electrophoresis. 2018 Mar 8;39(9-10):1208–15. doi: 10.1002/elps.201700437 (PMC6001557; doi:10.1002/elps.201700437)
Supplement: Supplementary file 1 — Supporting information [file ELPS-39-1208-s001.docx]

**Investigating capillary electrophoresis-mass spectrometry for the analysis of common post-translational modifications**

**Klaus Faserl^*^, Bettina Sarg^*^, Peter Gruber^§^, and Herbert H. Lindner^*¶^**

***Division of Clinical Biochemistry, Biocenter, Innsbruck Medical University, Innsbruck, Austria.**

**^§^Division of Medical Biochemistry, Biocenter, Innsbruck Medical University, Innsbruck, Austria.**

**^¶^**Corresponding author: Herbert Lindner Ph.D., Division of Clinical Biochemistry, Biocenter, Innsbruck Medical University, Innrain 80-82, A-6020 Innsbruck, Austria. Phone: 0043/512-9003-70310; Fax: 0043/512-9003-73300; E-mail: [Herbert.Lindner@i-med.ac.at](mailto:Herbert.Lindner@i-med.ac.at)

**MATERIALS AND METHODS**

**Enzymatic digestion:**

Extracted proteins from SILAC labeled PC-12 pheochromocytoma cells were split into 1.2 mg aliquots and solubilized in 400 µL ammonium bicarbonate buffer (100 mM, pH 8.0) each. Proteins were then reduced with 300 µL dithiothreitol (10 mM in 100 mM ammonium bicarbonate buffer) at 56°C for 30 min and alkylated with 300 µL iodoacetamide (55 mM in 100 mM ammonium bicarbonate buffer) at room temperature for 20 min. Samples were digested overnight by adding 30 µl trypsin (in 50 mM acetic acid) at 37°C. Protease / protein ratio was 1:80 (w/w). The final sample volume was 1ml.

**Phosphopeptide Enrichment:**

Phosphopeptide enrichment was performed by immobilized metal-affinity chromatography (IMAC) using PHOS-Select™ Iron Affinity Gel (Prod. No. P9740) and SigmaPrep Spin columns (Prod. No. SC1000) both obtained from Sigma-Aldrich (Vienna, Austria). Peptides were loaded onto the spin columns at a ratio of 120µg protein per 50µl IMAC beads according to *Thingholm et al.* [1]. Binding buffer was 30% ACN, 250 mM acetic acid and 400 mM ammonium hydroxide was used as elution solution.

**Phosphopeptide Fractionation by high pH RP-HPLC with subsequent LC-MS analysis:**

Phosphopeptide fractionation by high pH RP-HPLC was performed using an *XBridge BEH300 C18* 5 um 4.6x250 mm column (Waters, Eschborn, Germany) and abovementioned HPLC system. For HPLC solvents a 100 mM formic acid stock solution was adjusted to pH 10 using ammonium hydroxide. For Solvent A 10 ml of stock solution was diluted in 970 ml DI water and 20 ml acetonitrile, for solution B 10 ml stock solution was diluted (1:1, v/v) in DI water and brought to a final volume of 1 l with acetonitrile. The gradient started at 0% solvent B, was increased to 5% B in 10 min, to 35% B in 60 min, to 70% B in 15 min, and was held at 70% B for 10 min. Fraction collection was started 4 min after injection at 1 min intervals. A total of 70 fractions was collected, lyophilized and stored dry at -20°C. Previous to LC-MS analysis fractions were pooled by combining fraction 1, 24, 47; 2, 25, 48; and so on [2, 3]. LC-MS of pooled fractions was performed using an UltiMate 3000 nano-HPLC system (Dionex, Germering, Germany) coupled to the LTQ Orbitrap XL mass spectrometer via a nanospray ionization source. A homemade fritless fused silica capillary column (75 µm i.d. x 280 µm o.d.) packed with 10 cm of 3 µm reversed-phase C18 material (Reprosil) was used for peptide separation. Solvents for HPLC were 0.1% formic acid (solvent A) and 0.1% formic acid in 85% acetonitrile (solvent B). The gradient started at 4% solvent B and was increased linearly to 45% solvent B in 115 min and up to 100% B in 5 min. The flow rate was 250 nl*/*min.

**Phosphopeptide Fractionation by acidic RP-HPLC with subsequent CE-MS analysis:**

Peptide separation and analysis was performed as described in *Faserl et al*. with slight modifications [4]. Peptides resulting from phosphopeptide enrichment were loaded on a Beckman Gold HPLC system (Beckman Coulter, Brea, CA) and separated by reversed-phase chromatography using an NUCLEOSIL C18 120A 3µm 4.6x250mm column (Machery-Nagel, Düren, Germany) at a flow rate of 500 µL/min. HPLC solvents were 0.1% trifluoroacetic acid (solvent A) and 0.1% trifluoroacetic acid in 85% acetonitrile (solvent B). The gradient started at 4% solvent B for 14.5 min, was increased to 60% B in 90 min and up to 100% B in 4 min. Fraction collection was initiated 5 min after injection at 30 sec intervals. A total of 200 fractions were collected, lyophilized and stored dry at -20°C. Prior to CE-MS analysis, the peptides were dissolved in 10 µl ammonium acetate (50 mM, pH 4.0). For capillary electrophoresis a CESI 8000 (Sciex, Brea, CA) equipped with a bare fused silica capillary (total length: 90 cm, i.d.: 30 µm, o.d.: 150 µm) was coupled via an ESI module to a Thermo LTQ Orbitrap XL (Bremen, Germany). Prior to each analysis the system was rinsed with background electrolyte (10% acetic acid) for 3 min at 50 psi to refresh the buffer. Samples were injected by applying a pressure of 5 psi for 50 sec (38 nL) followed by an injection plug of BGE (5 psi for 5 sec). The separation was performed at +30 kV for 30 min.

**Mass Spectrometry**

The LTQ Orbitrap XL mass spectrometer was operating in data dependent mode to switch between MS and MS/MS acquisition. Survey full scan MS spectra were acquired in the Orbitrap with a resolution of R = 60,000 (at m/z =400) in profile mode after accumulation to an AGC target value of 1×10^6^ in the linear ion trap. MS/MS spectra were obtained in the linear ion trap (LTQ) using collision induced dissociation (CID). The 6 most intense precursors in CE-MS and the 10 most intense precursors in LC-MS were sequentially selected for MS/MS fragmentation. Parameters applied for fragmentation were: minimum signal required 1000; Isolation width (m/z) 2.0; activation time 30ms; normalized collision energy 35.0; and activation q of 0.25. MS/MS spectra were acquired in centroid mode with an AGC target value of 1×10^4^ and 100 ms maximum ionization time, respectively. Dynamic exclusion was set to 15 sec in CE-MS and 25 sec in LC-MS.

**Data analysis and peptide quantification.**

MaxQuant version 1.5.0.30 (ThermoScientific) was used for data analysis. Raw data obtained by CE-MS and LC-MS were searched against an *in silico* tryptic digest of the Uniprot rodent database (version June 2014; 24,157 sequences) using the search engine Andromeda implemented in MaxQuant software. The following settings were applied: Enzyme for protein cleavage was trypsin; three missed cleavages were allowed; precursor mass tolerance was set to 20 ppm in the first and 4.5 ppm in the main search the fragment mass tolerance was 20 ppm. Carbamidomethylation (C) was used as fixed modification; oxidation (M), N-terminal protein acetylation and phosphorylation (S T Y) were used as variable modifications; medium and heavy SILAC labels (K R) were used as labels. Solely peptides with a false discovery rate of less than 1% were accepted. For SILAC quantification, ^2^H_4_ lysine and ^13^C_6_ arginine were specified as medium labels (M), ^13^C_6_^15^N_2_ lysine and ^13^C_6_^15^N_4_ arginine were specified as heavy labels (H), respectively. Lysine (^12^C_6_^14^N_2_) and arginine (^12^C_6_^14^N_4_) were set as the light labels (L).

**References**

[1] Thingholm, T. E., Jensen, O. N., Robinson, P. J., Larsen, M. R., *Mol Cell Proteomics* 2008, *7*, 661-671.

[2] Wang, Y., Yang, F., Gritsenko, M. A., Wang, Y., Clauss, T., Liu, T., Shen, Y., Monroe, M. E., Lopez-Ferrer, D., Reno, T., Moore, R. J., Klemke, R. L., Camp, D. G., 2nd, Smith, R. D., *Proteomics* 2011, *11*, 2019-2026.

[3] Batth, T. S., Francavilla, C., Olsen, J. V., *J Proteome Res* 2014, *13*, 6176-6186.

[4] Faserl, K., Kremser, L., Muller, M., Teis, D., Lindner, H. H., *Anal Chem* 2015, *87*, 4633-4640.
